# Authentic Emotional Displays and Job Burnout Among Preschool Teachers: A Chain Mediation Model of Psychological Capital and Family–Work Conflict

**DOI:** 10.3390/bs16040483

**Published:** 2026-03-24

**Authors:** Xi Wang, Xingyu Duan, Jiajia Li, Chaopai Lin

**Affiliations:** 1 Department of Early Childhood Education, School of Education, Guangzhou University, Guangzhou 510006, China; wangxi@gzhu.edu.cn (X.W.); xingyu_duan@e.gzhu.edu.cn (X.D.); 2112508135@e.gzhu.edu.cn (J.L.); 2 Department of Early Childhood Education, School of Education, Central China Normal University, 1309 No. 3 Building of International Zone, No. 152 Luoyu Road, Hongshan District, Wuhan 430079, China

**Keywords:** authentic emotional displays, preschool teachers, job burnout, psychological capital, family work conflict

## Abstract

Emotional labor is inherently intensive in preschool teaching, and it is often conceptualized as resource loss, which may heighten burnout and hinder early childhood education quality-improvement initiatives. Accordingly, this study shifts attention to the resource-enhancing potential of authentic emotional displays and examines the mechanisms linking authentic displays to preschool teachers’ burnout, with psychological capital and family–work conflict as key mediators. Using cross-sectional survey data from 234 preschool teachers in Jiangxi Province, China, and structural equation modeling, we observed that authentic emotional displays were associated with lower burnout primarily through psychological capital, with an additional sequential association via psychological capital and family–work conflict, whereas the pathway through family–work conflict alone was not supported. These findings suggest a potential role of authentic emotional displays in relation to psychological resources and family-to-work spillover, extending emotional labor research and providing insights for interventions aimed at reducing preschool teacher burnout. The study challenges the prevailing resource loss perspective of emotional labor, clarifies psychological capital as a central mechanism linking authenticity to cross-domain strain and teacher well-being, and emphasizes the importance of interventions that strengthen psychological resources and boundary management.

## 1. Introduction

Job burnout is typically defined as a comprehensive psychological state formed by emotional exhaustion, depersonalization, and a sense of low accomplishment, which occurs as individuals experience prolonged work-related stress (Maslach & Jackson, 1981). The core dimensions of burnout reflect the psychological and emotional depletion individuals experience under prolonged work pressure, leading to the gradual depletion of emotional resources, a growing indifference toward others, and doubts about one’s self-worth. Previous theoretical research on burnout suggests that, according to Maslach’s theory, burnout is a progressive process, with emotional exhaustion typically emerging first, followed by depersonalization and reduced personal accomplishment. This progression indicates that burnout does not occur suddenly but is accumulated over time through ongoing stress. The main factors influencing burnout include the imbalance between the work demands individuals face and the resources they possess, with work pressure gradually building up.

The Conservation of Resources (COR) theory proposes that individuals are motivated to acquire, maintain, and protect resources they value, including material resources, social support resources, and energy resources (Hobfoll, 1989). When these resources are threatened, lost, or insufficient to meet environmental demands across life domains such as work and family, individuals experience stress responses. When such resource loss persists over time, individuals typically first exhibit emotional exhaustion as the core stress response, which may ultimately develop into burnout (Maslach & Jackson, 1981; Edelwich & Brodsky, 1980). Building on the COR framework, the Job Demands-Resources model distinguishes between job demands, such as workload and emotional regulation, which require sustained effort and are closely linked to emotional exhaustion, and job resources, including organizational support, autonomy, and social support, which are associated with achieving work goals, lower levels of depersonalization, and higher personal accomplishment (Demerouti et al., 2001). Individuals with higher levels of these job resources tend to report lower burnout levels. Based on the insights from the COR theory and the job demands-resources model (JD-R), it is evident that prolonged emotional regulation, heavy workloads, and conflicts between work and family can deplete resources, leading to emotional exhaustion and burnout. However, if individuals develop a resource accumulation model, they can build psychological resources, reduce depersonalization, and enhance personal accomplishment, helping them resist external pressures, such as family demands, and ultimately reduce burnout risk.

Teacher job burnout has increasingly been recognized not merely as an individual adaptation issue but as a systemic problem shaped by structural and organizational factors in educational settings (Modan, 2022), a phenomenon that has been further exacerbated in the post COVID-19 pandemic era (Zhou & Nanakida, 2023). A meta-analysis indicates that teachers worldwide experience substantial levels of burnout, largely associated with high emotional demands and classroom management challenges (Aloe et al., 2014). Similar patterns have also been observed among preschool teachers (Jeon et al., 2018). Existing surveys indicate that nearly half of preschool teachers in early childhood education contexts experience high levels of stress and job burnout, reflecting a persistent imbalance between emotional demands and available support (Modan, 2022). For preschool teachers, emotional regulation is a central aspect of their work, as they must manage their own emotions while supporting children’s social and emotional development (e.g., Cumming, 2017; Kwon et al., 2022; Schaack et al., 2020; L. Zhang et al., 2020). Compared with teachers at other educational levels, preschool teaching is a unique and demanding form of emotional labor, as children require continuous and close emotional support throughout the school day, teachers’ emotional and service behaviors are strictly regulated, and emotional labor is thus central to teaching in this context (Q. Zhang et al., 2020). Early studies often conceptualized emotional labor as a resource-depleting process, in which responding to organizational expectations for emotional expression consumes psychological resources, complicates the balance between work and family demands, and contributes to emotional strain, fatigue, and burnout (A. A. Grandey & Melloy, 2017; Brotheridge & Grandey, 2002; Deng et al., 2017; A. Grandey et al., 2012; Luo et al., 2025). However, research has also highlighted alternative strategies for emotional labor (A. Grandey et al., 2012), in which individuals’ emotional regulation aligns naturally with their inner feelings and requires less effort. Such strategies can protect against resource depletion, reduce the intrusion of other life domains on work resources, enhance teachers’ life satisfaction, and are associated with lower levels of burnout (Burić et al., 2021; Moran & Çoruk, 2021; Choi & Choi, 2025; Hao, 2024). However, the mechanisms through which this strategy influences burnout, by generating psychological resources and resisting the intrusion of resources from other domains, remain underexplored, particularly in the context of preschool education (Choi & Choi, 2025).

Therefore, this study examines job burnout among preschool teachers from a resource-enhancement perspective, focusing on how authentic emotional display may enhance psychological capital, reduce family–work conflict, and ultimately lower burnout. Previous research indicates that psychological capital (Luthans et al., 2006; Siu et al., 2014) and family–work conflict (Toprak et al., 2024) are important factors influencing teachers’ occupational well-being and burnout. By examining this sequential pathway, the study aims to clarify how workplace authenticity supports teachers’ psychological resources and overall well-being across both work and family domains.

### 1.1. The Relationship Between Authentic Emotional Displays and Job Burnout

Emotional labor, a concept first introduced by Hochschild (2012), refers to the process by which employees regulate their emotions to meet organizational display rules. Hochschild identified two primary strategies for emotional labor: surface acting and deep acting. Surface acting involves altering outward emotional expressions without changing internal feelings, whereas deep acting refers to efforts aimed at experiencing the emotions that are expected to be displayed. Subsequent research found that Hochschild’s framework interprets emotional labor through a resource depletion perspective, overlooking authentic emotional experience and the potential benefits of different regulation strategies (Diefendorff et al., 2005). Accordingly, Ashforth and Humphrey (1993) proposed genuine emotional expression as a third form of emotional labor, reflecting the alignment between employees’ true feelings and situationally appropriate emotional expression, a conceptualization that has been supported by subsequent research (Carey & Sutton, 2024; Lee et al., 2016; Wang et al., 2019). Grandey and colleagues later elaborated this concept, highlighting its low-effort, naturally aligned characteristics and its protective role in emotional labor (A. Grandey et al., 2012). Such alignment inherently reduces emotional dissonance, minimizes the strain associated with excessive regulation, and enables individuals to meet professional role expectations without substantial depletion of psychological resources (A. A. Grandey & Gabriel, 2015). This perspective has been further developed in the context of teachers’ emotional labor, with recent research examining how authentic emotional displays can align with contextual demands in teacher-student interactions (Choi & Choi, 2025; Hao, 2024). Emerging evidence suggests that the authenticity of teachers’ emotional displays can enhance their life satisfaction (Burić et al., 2021; Moran & Çoruk, 2021) and is associated with lower levels of job burnout (Choi & Choi, 2025). However, the underlying mechanisms linking authenticity to these outcomes remain underexplored, particularly in educational contexts.

Authenticity refers to the subjective experience where an individual’s actions and expressions align with their core values and identity (Lenton et al., 2013). When individuals perceive this alignment, they feel true to themselves, particularly when pursuing intrinsically valued goals (Ryan & Deci, 2000; Sheldon & Elliot, 1998). Research differentiates between trait authenticity, which represents a stable individual disposition, and state authenticity, a context-dependent experience that varies across situations (Lenton et al., 2013; Wood et al., 2008; Van den Bosch & Taris, 2014). In organizational contexts, state authenticity is especially relevant, as employees often need to regulate their emotions and behaviors to meet workplace expectations (Ashforth & Humphrey, 1993; Xie & Yang, 2025). According to Self-Determination Theory, authenticity reflects self-determined motivation, where behaviors driven by internal values reduce emotional dissonance and make emotional regulation more autonomous and less psychologically demanding (A. A. Grandey, 2000). Research has shown that the level of authenticity in the workplace plays a crucial role in preventing job burnout (Maslach & Leiter, 2016). First, when employees feel authentic, their emotional expressions are more in tune with their internal states. This alignment helps maintain genuine interpersonal connections and reduces emotional detachment, which may lower the likelihood of depersonalization (Maslach et al., 2001). Second, authenticity supports intrinsic motivation, allowing individuals to engage more fully in their work activities. This increases persistence when facing challenges and enhances their sense of competence and accomplishment (Ryan & Deci, 2000; White et al., 2024). Lastly, authentic emotional regulation requires fewer psychological resources than externally imposed emotional control, thereby reducing emotional exhaustion over time (A. A. Grandey & Gabriel, 2015; Schaufeli & Bakker, 2004). As a result, authenticity serves as a protective factor against multiple dimensions of burnout (Maslach & Leiter, 2016). Preschool teaching is a typical form of emotional labor, in which kindergartens script interactions among teachers, children, and families and tightly regulate teachers’ emotional and service behaviors (L. Zhang et al., 2020). Although existing research has found that growing attention has been paid to the role of authentic emotional displays in teaching (Keller & Becker, 2021; Zheng et al., 2024), and has identified an impact of authentic emotional displays on the mental health and job satisfaction of teachers (Taxer & Frenzel, 2015; H. Yin et al., 2019), most empirical studies have focused on primary and secondary school teachers, with relatively fewer studies in the context of preschool education (Keller & Becker, 2021; H. Yin et al., 2017; Zheng et al., 2024). Based on the literature above, authentic emotional displays, as a beneficial self-consistency based emotion regulation strategy for preschool teachers, tend to be linked with lower levels of job burnout. Therefore, we propose Hypothesis 1: Preschool teachers’ authentic emotional displays are negatively associated with job burnout.

### 1.2. The Mediating Role of Psychological Capital Between Authentic Emotional Displays and Job Burnout

Psychological capital refers to an individual’s positive psychological state of development and comprises four dimensions: hope, resilience, optimism, and self-efficacy (Luthans et al., 2004). Emotion regulation strategies and self-consistency are important psychological mechanisms influencing the formation and accumulation of psychological capital. Authentic emotional displays may provide an effective, low-cost regulation strategy by reducing the need for effortful suppression or manufacturing. When teachers’ expressions align with their genuine experiences and self-concept, they appear more sincere and are more likely to receive understanding and support from students, parents, and colleagues, strengthening social support (Cohen & Wills, 1985) and enhancing goal confidence and self-efficacy (Bandura & Wessels, 1997). At the same time, authentic emotional displays enhance individuals’ ability to recover from challenges, thus reinforcing resilience and optimistic tendency (Avey et al., 2010; Gross, 1998). This consistency reduces cognitive dissonance and self-alienation, which in turn lowers the loss of psychological energy and helps maintain positive psychological states over time (Ashforth & Humphrey, 1993; Kernis & Goldman, 2006). Drawing on self-determination theory, authentic emotional displays that are aligned with internal values and enacted autonomously can support psychological capital by strengthening self-efficacy (Ryan & Deci, 2000). Previous studies have found that authentic emotional displays in organizational contexts promote beneficial outcomes (Fredrickson, 2001; Wahid et al., 2023). Scott et al. (2020) found that the natural expression of genuinely felt positive emotions involves less emotional exhaustion than surface acting and is associated with higher job satisfaction. Aw et al. (2020) further showed that authentic emotional displays not only enhance subjective job performance but also lead to improvements in objective performance indicators.

Overall, authentic emotional displays align felt and expressed emotions, reducing emotional dissonance and conserving psychological resources that support preschool teachers’ long-term occupational health. The costs of emotional labor are not inevitable; when teachers strategically adopt authentic emotional displays, emotion regulation can generate positive psychological resources and sustain internal motivation for career development. Based on this theoretical and empirical foundation, this study proposes Hypothesis 2: psychological capital serves as a mediator in the relationship between authentic emotional displays and job burnout among preschool teachers. Specifically, authentic emotional displays are positively related to psychological capital, which is negatively related to job burnout.

### 1.3. The Mediating Role of Family–Work Conflict Between Authentic Expressional Displays and Job Burnout

Work-family conflict (WFC) refers to the mutual interference and resource conflict that arise when individuals attempt to fulfill work and family roles simultaneously under conditions of limited time, energy, or emotional resources (Greenhaus & Beutell, 1985). It includes work-to-family conflict, in which work demands interfere with family roles, and family-to-work conflict, in which family responsibilities and pressures spill over into the work context (Frone et al., 1992; Netemeyer et al., 1996). Given the bidirectional nature of work–family conflict, work-to-family conflict primarily affects family-related outcomes and has relatively weak relevance for predicting job burnout (Michel et al., 2011; Byron, 2005). In contrast, individuals in the workplace may be influenced by family-to-work conflict, as stress, emotional strain, and role tension arising in the family domain can spill over into the work context through emotional, cognitive, and behavioral processes (Allen et al., 2000). In this process, family demands consume individuals’ time and emotional energy, reducing the resources available for work engagement (Bao et al., 2025; Greenhaus & Beutell, 1985). In early childhood education, family-related demands particularly deplete the emotional and cognitive resources required for high-quality interactions with children, making family-to-work conflict a significant predictor of work-related stress (Frone et al., 1992; Netemeyer et al., 1996; Bao et al., 2025).

From the perspective of Self-Determination Theory (SDT), authentic emotional displays typically stem from autonomous regulation, meaning that individuals’ behaviors align with their intrinsic values and interests (Ryan & Deci, 2000). This self-consistency between internal states and external expressions reduces role conflict and conserves psychological resources, allowing family-related demands to be integrated rather than disrupt work, thereby mitigating family-to-work conflict (Kernis, 2003; Ryan & Deci, 2000; Q. Zhang et al., 2020). In addition, authentic emotional displays fosters trust and positive interactions, improving relationships with colleagues and parents and forming stable social support networks, which serve as emotional and cognitive buffers against family stress spillover (Cohen & Wills, 1985). In emotionally demanding early childhood education settings, these resources enable teachers to better manage family-to-work interference, alleviating its negative impact on work engagement and occupational burnout (Li & Liu, 2025).

Although work and family draw on overlapping personal resources and family-related strain can spill over into the work domain, persistent family demands are commonly associated with emotional resource depletion and higher levels of job burnout (Grzywacz & Marks, 2000; Ten Brummelhuis & Bakker, 2012). However, higher levels of authentic emotional displays are associated with more self-consistent emotion regulation, which is more likely to be experienced as a personal emotional resource rather than an additional regulatory burden (Choi & Choi, 2025). Such authenticity is linked to reduced accumulation of family-to-work conflict and lower emotional exhaustion. Based on this, Hypothesis 3 proposes that authentic emotional displays are indirectly associated with preschool teachers’ job burnout through family–work conflict. Specifically, preschool teachers with higher levels of authentic emotional displays tend to engage in more self-consistent emotion regulation, which is associated with lower emotional depletion in the presence of family interference and, in turn, lower levels of job burnout.

### 1.4. The Chain-Mediated Relationship Between Psychological Capital and Family–Work Conflict

Psychological capital is a core personal resource that supports adaptive functioning under stress (Luthans et al., 2006). Situational stress can erode personal resources over time, and in the work-family interface, time and energy depleted in one domain can carry over across boundaries to shape functioning in the other (Ten Brummelhuis & Bakker, 2012). Across the work-family interface, psychological capital may be depleted as individuals manage demands in both domains (Fu & Li, 2025), and stronger boundary dynamics can constrain spillover by limiting cross-domain strain transmission. Conservation of Resources theory posits that individuals are motivated to acquire, protect, and accumulate valuable resources, and that the extent of resource possession influences their ability to cope with stress (Hobfoll, 1989, 2002). Individuals with higher levels of psychological capital typically demonstrate more positive cognitive styles and stronger coping capacities, enabling them to manage the multiple demands arising from both work and family domains more effectively (Bai et al., 2025). A comprehensive review of dispositional traits and work-family conflict found that personal resources such as positive affect, self-efficacy, and optimism are significantly associated with lower levels of work-family conflict (Allen et al., 2012; Yan et al., 2024). Individuals with high levels of role coordination tend to experience less strain associated with multiple role demands (Barrick & Mount, 1991). Accordingly, psychological capital helps individuals integrate work and family role demands more effectively and reduces the negative interference of family demands with work (Yan et al., 2022).

For preschool teachers, authentic emotional display helps maintain a positive psychological state and provides a foundation for the development and accumulation of psychological capital (Karatepe & Karadas, 2014). Through authentic emotional display, individuals can activate intrinsic motivation and reduce resource depletion caused by external pressures (Ryan & Deci, 2000). Psychological capital is not only fostered by positive emotional experiences but also shapes individuals’ cognitive appraisals of stressful events. Based on Conservation of Resources theory, individuals with higher psychological capital are more likely to perceive family-related stressors as manageable challenges rather than uncontrollable threats, thereby experiencing lower levels of family-to-work conflict (Allen et al., 2012) and ultimately reduced job burnout. Based on these theoretical arguments, the present study proposes Hypothesis 4: psychological capital and family–work conflict play a serial mediating role in the relationship between authentic emotional display and job burnout. Specifically, authentic emotional display tends to be associated with higher levels of psychological capital, which is further related to lower family-to-work conflict and, in turn, lower levels of job burnout among preschool teachers.

### 1.5. Summary

In summary, this study examines the associations of authentic emotional displays with preschool teachers’ psychological resources and their interactions across contexts, viewing authentic emotional displays as a form of internally consistent emotion regulation linked to positive psychological resources, and offering an alternative perspective to the traditional view of emotional labor as a source of resource loss. At the work environment level, authentic emotional displays are related to lower emotional regulation demands, greater emotional consistency, and the maintenance of psychological integrity, which are in turn associated with lower levels of job burnout among preschool teachers. At the family–work interface, authentic emotional displays are associated with lower levels of family-to-work strain by limiting emotional and psychological resource loss across contexts, and are further linked to reduced family–work conflict. Taken together, this perspective enriches the understanding of the potential functions of emotional labor and offers theoretical insights into promoting emotional well-being and sustainable career development among preschool teachers.

## 2. Methods

### 2.1. Participants

Participants were 234 preschool teachers (94.4% female) from Jiangxi Province, China. Their ages ranged from 17 to 58 years (M = 30.0, SD = 9.15). A few participants were just under 18, reflecting the rapid expansion of early childhood education in China and the inconsistent application of entry requirements and teacher qualification regulations across regions. On average, participants had 7.69 years of teaching experience (SD = 8.12), with individual experience ranging from 0.4 to 38 years. Approximately 67.9% of teachers worked in rural areas, and 29.1% were based in urban settings. Regarding educational background, 62.4% held associate degrees, 29.5% held bachelor’s degrees or above, and 7.7% had completed high school or less. In terms of teaching roles, 63.7% were head teachers, while 30.3% served as assistant teachers. Additionally, 56.0% of participants had tenured positions.

### 2.2. Procedures

A cross-sectional survey was conducted among early childhood educators involved in a continuous professional development program in Jiangxi Province, China. Data collection occurred between March and June 2023. Preschool teachers were recruited through their respective early childhood education institutions participating in the larger teacher-training project initiated by the Jiangxi Provincial Education Department. The surveys were administered electronically via Wenjuanxing (https://www.wjx.cn), a widely used online data collection platform in China. Informed consent was obtained electronically from all participating teachers prior to the start of the survey, ensuring their voluntary participation. All teachers enrolled in the professional development program completed the survey, resulting in a response rate of 100%. Given that participation was coordinated through the training program institutions, the risk of nonresponse bias was minimal. It should be noted that participants were recruited through a convenience sample from the professional development program, which may introduce selection bias and limit the generalizability of the findings to the broader population of preschool teachers. The present study utilized data from 234 participants who completed the survey. The research protocol was approved by the Institutional Review Board of the corresponding author’s institution.

### 2.3. Measures

#### 2.3.1. Authentic Emotional Displays

Authentic emotional displays were measured using the three-item Expression of Naturally Felt Emotions subscale adapted from the Emotional Labor Scale developed by Diefendorff et al. (2005). Items were rated on a 5 point Likert scale ranging from 1 (Strongly disagree) to 5 (Strongly agree), with higher scores indicating higher levels of authentic emotional displays. This dimension was specifically selected for its appropriateness in early education settings, as teachers are typically encouraged to display authentic emotions to foster genuine teacher-child interactions conducive to children’s socioemotional development. A sample item includes, “The emotions I express to children are genuine”. In the present study, internal consistency was acceptable (Cronbach’s α = 0.73, Composite reliability = 0.73). Confirmatory factor analysis indicated saturated model fit (χ^2^ = 0, df = 0, CFI = 1.00, TLI = 1.00, RMSEA = 0.00, SRMR = 0.00), with all standardized loadings significant.

#### 2.3.2. Psychological Capital

Psychological capital was assessed using the 24-item Psychological Capital Questionnaire developed by Luthans et al. (2006). Items were rated on a 5 point Likert scale ranging from 1 (Strongly disagree) to 5 (Strongly agree), with higher scores indicating higher psychological capital. The scale includes four dimensions: Self-Efficacy (e.g., “I feel confident analyzing a long-term problem to find a solution”), Hope (e.g., “At this time, I am meeting the work goals that I have set for myself”), Resilience (e.g., “I usually take stressful things at work in stride”), and Optimism (e.g., “I always look on the bright side of things regarding my job”). The internal consistency for the total scale in the current sample was high (Cronbach’s α = 0.89, Composite reliability = 0.88).

#### 2.3.3. Work-Family Conflict

The Family-to-Work Conflict dimension from Netemeyer et al.’s (1996) Work-Family Conflict Scale was utilized, given the study’s focus on examining how preschool/teachers’ familial responsibilities influence their occupational roles and performance. This dimension comprises 5 items. Items were rated on a 5-point Likert scale ranging from 1 (Strongly disagree) to 5 (Strongly agree), with higher scores indicating greater family to work conflict. A representative item from this dimension includes, “Family-related strain interferes with my ability to perform job-related duties.” This dimension demonstrated good internal consistency in the current study (Cronbach’s α = 0.86). This dimension demonstrated good internal consistency (Cronbach’s α = 0.86, composite reliability = 0.86). Confirmatory factor analysis indicated good model fit in the current sample, χ^2^(5) = 9.755, *p* < 0.001; CFI = 0.99; TLI = 0.98; RMSEA = 0.06; SRMR = 0.02.

#### 2.3.4. Teacher Burnout

Teacher burnout was measured using the Maslach Burnout Inventory–Educator’s Survey (MBI-ES; Maslach & Jackson, 1981), validated for preschool contexts by Aboagye et al. (2018). The inventory consists of 22 items across three subscales: Emotional Exhaustion (e.g., “I feel used up at the end of the workday”), Depersonalization (e.g., “I feel I treat students as if they were impersonal objects”), and Personal Accomplishment (e.g., “I have accomplished many worthwhile things in this job”). Items were rated on a 5-point scale ranging from 1 (Never) to 5 (Every day), higher scores indicate higher levels of burnout. The total scale yielded high internal reliability (Cronbach’s α = 0.88, Composite reliability = 0.88).

#### 2.3.5. Covariates

Covariates included teaching experience, educational background (1 = High school or below, 2 = Associate, 3 = Bachelor or above, since only one participant had a postgraduate degree, this category was collapsed into “bachelor’s degree or above.”), and tenured status, given their established associations with psychological outcomes, strategies for managing work-family conflict, and burnout among educators (Chang, 2009; Skaalvik & Skaalvik, 2010). These variables were thus controlled for in subsequent analyses to enhance interpretive clarity.

### 2.4. Analytic Strategy

Structural equation modeling (SEM) was employed to test the mediating roles of psychological capital and family-to-work conflict. Analyses were conducted in two stages. First, descriptive analyses, including assessments of missing data patterns, were performed using R (version 4.4.1; R Foundation for Statistical Computing, Vienna, Austria). The proportion of missing data in the current sample was 4.7%. Little’s Missing Completely at Random (MCAR) test revealed a non-significant result, χ^2^(139) = 160, *p* = 0.112. A dummy indicator capturing any missingness on the focal measures was regressed on observed demographic and job-related characteristics using logistic regression; none of the predictors were significant (all *p*s > 0.10), suggesting no systematic differences between respondents with versus without missing data. Therefore, Full Information Maximum Likelihood (FIML) was used to handle missing data in the subsequent analyses.

Second, mediation analyses were conducted using Mplus (version 8.11; Muthén & Muthén, 2017, Los Angeles, CA, USA). To ensure the stability of mediation estimates, 95% confidence intervals (CIs) were computed using the bias-corrected bootstrap method with 5000 resamples. To reduce estimation complexity due to the multidimensional nature of some measures, a parceling approach was applied. Specifically, for the psychological capital and burnout measures—the mean scores of each dimension served as parcel indicators for their corresponding latent constructs. Authentic emotional displays and family-to-work conflict, measured as unidimensional constructs, were modeled using their original item scores as latent indicators.

Model fit was evaluated based on the following commonly accepted criteria: Root Mean Square Error of Approximation (RMSEA ≤ 0.08; Hu & Bentler, 1999), Comparative Fit Index (CFI ≥ 0.90 acceptable, ≥0.95 good fit; Marsh et al., 2004; Hu & Bentler, 1999), Tucker–Lewis Index (TLI ≥ 0.90 acceptable, ≥0.95 good fit; Marsh et al., 2004; Hu & Bentler, 1999), and Standardized Root Mean Square Residual (SRMR ≤ 0.08; Hu & Bentler, 1999).

## 3. Results

### 3.1. Test of Common Method Biases

To assess potential common method bias, Harman’s single factor test was conducted using an unrotated exploratory factor analysis across all measurement items. The results indicated that 19 components had eigenvalues greater than 1, suggesting that variance was distributed across multiple factors rather than dominated by a single latent source. Importantly, the first component accounted for 22.157% of the total variance, which is below commonly cited thresholds used to flag serious common method concerns. These findings suggest that common method bias is unlikely to pose a substantial threat to the validity of our results.

### 3.2. Preliminary Analyses

Bivariate correlations among key study variables presented in Table 1 revealed significant associations consistent with theoretical expectations. Specifically, Authentic emotional displays negatively correlated with burnout (*r* = −0.38, *p* < 0.001), positively correlated with psychological capital (*r* = 0.46, *p* < 0.001) and negatively correlated with family-to-work conflict (*r* = −0.16, *p* < 0.05). Additionally, both mediators exhibited significant relationships with burnout. Psychological capital showed a strong negative correlation with burnout (*r* = −0.63, *p* < 0.001), whereas family-to-work conflict was positively correlated with burnout (*r* = 0.48, *p* < 0.001).

### 3.3. The Mediating Effect of Psychological Capital and Work-Family Conflict

Before the formal hypothesis testing, we conducted an exploratory comparison of alternative models to evaluate the robustness of the proposed serial mediation structure given the cross-sectional nature of the data (Table 2). We compared the hypothesized serial mediation model with two nested constrained models and one reversed ordering model: (a) a parallel mediation model constraining the serial link psychological capital → family-to-work conflict to zero, (b) a pure-chain specification constraining the nonserial paths authentic emotional displays → psychological capital and family-to-work conflict → burnout to zero, and (c) a fully reversed ordering model (burnout → family-to-work conflict → psychological capital → authentic emotional displays). The nested comparisons showed that both constrained models fit significantly worse than the baseline, supporting the incremental contribution of the theorized serial structure. For the fully reversed model, global fit is statistically equivalent to the baseline; however, only burnout → psychological capital, burnout → family-to-work conflict, and psychological capital → authentic emotional displays were significant, whereas the remaining links were nonsignificant, offering limited support for a coherent reversed chain. Overall, these comparisons supported retaining the theorized serial mediation model for subsequent interpretation of the structural paths.

**Figure 1:**
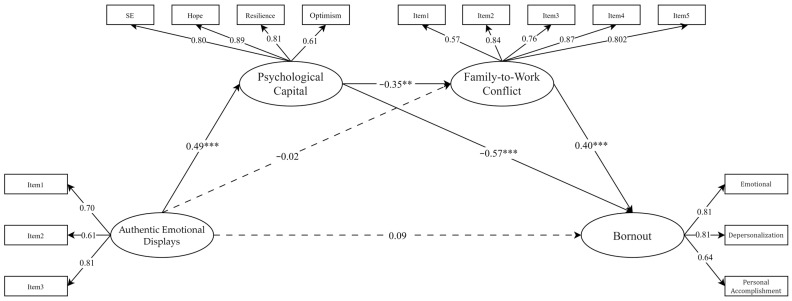
Mediation model for PS and FWC between AED and Burnout. Note. Teacher’s teaching years, Education background, and tenured status were included as covariates. Solid lines indicate significant paths (*p* < 0.05), and dashed lines indicate nonsignificant paths (*p* > 0.05). ** *p* < 0.01, *** *p* < 0.001.

As shown in Table 2, the mediation SEM model showed marginally acceptable fit. Figure 1 presents the standardized path coefficients. Authentic emotional displays was positively associated with psychological capital (β = 0.49, *p* < 0.001) but was not directly associated with family-to-work conflict (β = −0.02, *p* = 0.86) or burnout (β = 0.09, *p* = 0.38). Psychological capital negatively predicted burnout (β = −0.57, *p* < 0.001) and family-to-work conflict (β = −0.35, *p* = 0.003), and family-to-work conflict was positively related to burnout (β = 0.40, *p* < 0.001). Among the covariates, teaching experience was positively associated with psychological capital (β = 0.39, *p* < 0.001) and family-to-work conflict (β = 0.20, *p* = 0.032), with all other covariate effects nonsignificant.

The bootstrapped mediation analysis (5000 resamples) further revealed significant effects (Table 3). The total effect of authentic emotional displays on burnout was significant (95% CI = [−0.49, −0.04]), whereas the direct effect was not (95% CI = [−0.12, 0.29]). These results support Hypothesis 1, indicating a negative association between authentic emotional displays and burnout. The direct effect of authentic emotional displays on burnout became non-significant when psychological capital and family-to-work conflict were included, indicating that the association between authentic emotional displays and burnout was primarily transmitted through psychological capital and family-to-work conflict. The total indirect effect was significant (95% CI = [−0.51, −0.21]). Given that the direct and indirect effects were opposite in sign, we report the relative contribution of each specific indirect effect as a proportion of the total indirect effect. Psychological capital accounted for the majority of the total indirect effect (77.8%, 95% CI = [−0.43, −0.15]), followed by the sequential pathway through psychological capital and family-to-work conflict (19.4%, 95% CI = [−0.13, −0.03]); the indirect effect via family-to-work conflict alone was not significant (95% CI = [−0.08, 0.06]).

## 4. Discussion

Existing research on emotional labor often directly associates it with emotional exhaustion and psychological resource loss, where emotional labor is typically seen as a highly draining job demand (Jeung et al., 2018). However, it is important to note that the pathways through which different types of emotional labor affect psychological mechanisms and outcome variables are not consistent (A. Grandey et al., 2012). In this study, emotional labor specifically refers to authentic emotional displays, where preschool teachers express their authentic emotions in educational contexts, rather than intentionally suppressing or masking their emotions to conform to external norms.

The statistical findings indicate that the total effect of authentic emotional displays on burnout was negative and significant, supporting Hypothesis 1. However, this association became nonsignificant after psychological capital and family-to-work conflict were included in the model. Mediation structural equation modeling further showed that authentic emotional displays were not directly associated with job burnout but were indirectly associated with burnout through psychological capital, supporting Hypothesis 2. In addition, a significant sequential indirect effect through psychological capital and family-to-work conflict was observed, supporting Hypothesis 4. Notably, the model fit of the structural model was acceptable, though not optimal. This mechanism is consistent with the self-consistency perspective (Sheldon et al., 2023; Sheldon & Elliot, 1998), which posits that when individuals’ emotional experiences align with their outward emotional expressions, it helps reduce internal emotional conflict and psychological depletion, thereby promoting the accumulation of psychological resources. Landa and English (2022) also found that the authenticity of emotional expression predicted higher levels of positive emotions and lower levels of negative emotions. Studies on teachers have similarly found that authentic emotional displays are associated with greater psychological coherence and higher levels of psychological capital, which are linked to more adaptive responses to job demands and a lower risk of job burnout, suggesting a potential protective role against occupational stress (Taxer & Gross, 2018). This study extends the explanatory framework of emotional labor research, advancing the understanding of its internal heterogeneity, and emphasizes that emotional labor is a multifaceted process. Future research should distinguish between different forms of emotional labor and their resource implications, forming an important distinction from prior research that mainly focuses on surface acting or deep acting perspectives. Particularly for the group of preschool teachers, traditional research has often emphasized the exhausting impact of emotional labor on teachers’ psychological resources and self-consistency (Côté, 2005; Ashforth & Humphrey, 1993; Carey & Sutton, 2024). Authentic emotional displays may be linked to lower emotional regulation effort and the preservation of self control resources and psychological capital, which are important emotional resources for resilience and coping with daily demands (A. Grandey et al., 2012; Muraven et al., 2006; Rosco et al., 2021).

Moreover, preschool teachers’ authentic emotional displays show no direct association with job burnout through family–work conflict but are indirectly associated with it through psychological capital, reflecting a sequential association linking psychological capital, family–work conflict, and job burnout. This result suggests that the association between authentic emotional displays and job burnout is sequential and dependent on psychological resources. Previous studies have established a link between authenticity at work and job satisfaction, yet often fail to incorporate the work-family boundary into their analysis (Mellors & Gaspar, 2025). Based on this, our study expands the perspective and reveals the crucial role of psychological capital in linking authentic emotional displays, family–work conflict, and job burnout (Wayne et al., 2019; White et al., 2024). According to Boundary theory (Clark, 2000), work and family are conceptualized as two relatively independent domains, with individuals managing the boundaries between them to reduce work-family conflict and maintain effective role functioning. This perspective suggests that authentic emotional displays may be related to stronger psychological resources, which could be relevant for how preschool teachers navigate demands across work and family domains and might be associated with reduced spillover of family-related stress into the work domain, and, in turn, with lower levels of burnout (Glavin & Schieman, 2012; Žiedelis et al., 2023). Self-determination theory posits that when contexts support autonomy and individuals act in ways aligned with their values and goals, they are more likely to feel connected and effective in their roles, thereby enhancing role satisfaction and engagement and reducing the risk of job burnout (Ryan & Deci, 2000; Kuvaas et al., 2017).

This study integrates authentic emotional displays, psychological capital, family–work conflict, and job burnout within an integrative framework, and examines an indirect pathway in which authentic emotional displays are associated with stronger psychological resources, lower cross-domain conflict, and, in turn, reduced burnout. The findings not only challenge the traditional view that emotional labor inevitably depletes resources, but also broaden our understanding of the roles of psychological capital and work-family conflict in the context of emotional labor. At the same time, our study found that preschool teachers’ teaching experience is negatively correlated with job burnout, which is consistent with previous research (Choi & Choi, 2025).

## 5. Practical Implications

This study finds that preschool teachers’ authentic emotional displays are indirectly associated with lower job burnout through psychological capital and reduced family–work conflict. These findings suggest that kindergarten leaders and educational administrators could consider fostering work environments that support authentic emotional displays (Collie et al., 2012). For example, organizational emotional norms may be adjusted to encourage preschool teachers to respond to young children’s emotions in natural, sincere, and warm ways, which might help reduce internal emotional conflict and support psychological resources. In addition, differentiated support may be considered to address the varying needs of preschool teachers. Kindergarten management might explore flexible work arrangements and programs for psychological resource development. Meanwhile, less experienced teachers could potentially benefit from targeted interventions, such as psychological capital enhancement programs and emotion regulation skills training, which may help support the development of stable psychological resource reserves. Overall, preschool teacher training and management practices may consider emphasizing internal psychological mechanisms and emotional resource management, potentially promoting higher quality professional development and supporting occupational health among preschool teachers.

## 6. Limitations and Future Research

This study examines the associations among authentic emotional displays, psychological capital, family–work conflict, and job burnout, offering insights into the emotional labor and resource accumulation processes of preschool teachers. Nevertheless, several limitations should be considered when interpreting the findings. Firstly, this study employs a cross-sectional design. As Cole and Maxwell (2003) pointed out, testing mediation mechanisms and sequential pathways ideally requires longitudinal data. Therefore, causal interpretations regarding how authentic emotional displays alleviate job burnout through psychological capital should be made with caution. Future research could employ longitudinal or experimental designs to better capture the dynamic processes of preschool teachers’ authentic emotional displays, psychological capital, and job burnout over time. Based on this, future studies should pay greater attention to organizational-level factors, including institutional climate, administrative support, teacher collaboration, work distribution, and policy variations, to better understand the mechanisms underlying preschool teachers’ psychological resource development and job burnout. The study sampled preschool teachers from a single Chinese province, which may limit generalizability. Related cultural norms emphasizing harmony, authority, and emotional restraint may influence both emotional labor demands and self-reports of exhaustion and authenticity (H. B. Yin & Lee, 2012), while culturally influenced response styles may further affect measurement validity (H. B. Yin & Lee, 2012; Johnson et al., 2005). Future research should include more diverse samples and consider complementary methods or statistical approaches to account for these culturally shaped response tendencies. Thirdly, while existing theories and research align with the findings of this study, there is still a lack of more direct empirical evidence to support these interpretations. Future studies could adopt longitudinal or multi-wave designs to more systematically explore the proposed serial mediation pathways. In addition, incorporating complementary methods, such as interviews or observations, may help enhance the robustness of the findings. Finally, future studies could include factors such as emotional labor norms, organizational support, and resource allocation in kindergartens as control variables to more systematically examine the mechanisms linking authentic emotional displays to preschool teachers’ occupational mental health.

## 7. Conclusions

This study systematically reveals that authentic emotional displays are indirectly associated with job burnout through a sequential pathway involving psychological capital and family–work conflict in the high emotional labor context of preschool teachers. This challenges the traditional view of emotional labor, which primarily emphasizes its depleting effects. Further analysis indicates that authentic emotional displays are associated with higher psychological capital, which is in turn related to lower family-to-work conflict, forming a sequential cross-domain association with job burnout. This mechanism extends emotional labor theory from work performance and resource depletion to cross-domain psychological health, offering insights into psychological capital development and informing interventions to support teachers’ occupational well-being.

## Figures and Tables

**Table 1:** Means, standard deviations, and bivariate correlations for variables.

Variable	M	SD	1	2	3	4	5	6	7
1. Teaching Years	7.69	8.12	–						
2. Educational Background ^a^	2.22	0.57	0.22 ***	–					
3. Tenure Status ^b^	1.43	0.50	−0.09	−0.43 ***	–				
4. Authentic Emotional Displays	3.92	0.68	0.11	0.06	0.08	–			
5. Psychological Capital	3.69	0.46	0.40 ***	−0.02	0.11	0.46 ***	–		
6. Family-to-Work conflict	1.84	0.77	0.08	0.15 *	−0.06	−0.16 *	−0.24 ***	–	
7. Burnout	2.39	0.59	−0.24 ***	0.00	−0.14	−0.38 ***	−0.63 ***	0.48 ***	–

*Note*. ^a^ Education background: 1 = High school or below, 2 = Associate, 3 = Bachelor or above. ^b^ Employment status: 1 = Tenured, 2 = Non-tenured. * *p* < 0.05, *** *p* < 0.001.

**Table 2:** Model fit indices for the comparative models.

Models	χ^2^(df)	CFI	TLI	RMSEA	SRMR	Δχ^2^	*p*
Baseline model	244.39 (117)	0.92	0.90	0.07	0.06	—	—
Parallel model	255.17 (118)	0.92	0.90	0.07	0.08	10.78 (1)	0.001
Pure-chain model	282.63 (119)	0.90	0.88	0.08	0.09	38.24 (2)	<0.001
Reversed model	244.39 (117)	0.92	0.90	0.07	0.06	0.00 (0)	—

**Table 3:** The bootstrap confidence interval and standardized effect size of the mediation model.

Effect	Path	Effect	SE	95% CI	Ratio
Total	AED → BO	**−0.27**	**0.12**	**[−0.49, −0.04]**	—
Total Indirect		**−0.36**	**0.08**	**[−0.51, −0.21]**	—
Specific Indirect	AED → PC → BO	**−0.28**	**0.07**	**[−0.43, −0.15]**	77.8%
	AED → WFC → BO	−0.01	0.04	[−0.08, 0.06]	2.8%
	AED → WFC → PC → BO	**−0.07**	**0.02**	**[−0.13, −0.03]**	19.4%
Direct	AED → BO	0.09	0.1	[−0.12, 0.29]	—

*Note*. AED = Authentic Emotional Displays, PC = Psychological Capital, WFC = Family-to-Work conflict, BO = Burnout. The bold statistics are significant based on the bias-corrected Bootstrapped 95% CI.

## Data Availability

The data that support the findings of this study are available on request from the corresponding author. The data are not publicly available due to privacy or ethical restrictions.

## References

[B1-behavsci-16-00483] Aboagye M. O., Qin J., Qayyum A., Antwi C. O., Jababu Y., Affum-Osei E. (2018). Teacher burnout in pre-schools: A cross-cultural factorial validity, measurement invariance and latent mean comparison of the Maslach Burnout Inventory, Educators Survey (MBI-ES). Children and Youth Services Review.

[B2-behavsci-16-00483] Allen T. D., Herst D. E., Bruck C. S., Sutton M. (2000). Consequences associated with work-to-family conflict: A review and agenda for future research. Journal of Occupational Health Psychology.

[B3-behavsci-16-00483] Allen T. D., Johnson R. C., Saboe K. N., Cho E., Dumani S., Evans S. (2012). Dispositional variables and work–family conflict: A meta-analysis. Journal of Vocational Behavior.

[B4-behavsci-16-00483] Aloe A. M., Amo L. C., Shanahan M. E. (2014). Classroom management self efficacy and burnout: A multivariate meta analysis. Educational Psychology Review.

[B5-behavsci-16-00483] Ashforth B. E., Humphrey R. H. (1993). Emotional labor in service roles: The influence of identity. Academy of Management Review.

[B6-behavsci-16-00483] Avey J. B., Luthans F., Smith R. M., Palmer N. F. (2010). Impact of positive psychological capital on employee well-being over time. Journal of Occupational Health Psychology.

[B7-behavsci-16-00483] Aw S. S. Y., Ilies R., De Pater I. E. (2020). Dispositional empathy, emotional display authenticity, and employee outcomes. Journal of Applied Psychology.

[B8-behavsci-16-00483] Bai B., Ma R., Rehima J. (2025). Exploring the impact of psychological capital and work family conflict on stress regulation and success in competitive athletes. Frontiers in Psychology.

[B9-behavsci-16-00483] Bandura A., Wessels S. (1997). Self-efficacy: The exercise of control.

[B10-behavsci-16-00483] Bao X., Dong J., Guo J. (2025). Family-to-work conflict and innovative work behavior among university teachers: The mediating effect of work stress and the moderating effect of gender. Behavioral Sciences.

[B11-behavsci-16-00483] Barrick M. R., Mount M. K. (1991). The big five personality dimensions and job performance: A meta-analysis. Personnel Psychology.

[B12-behavsci-16-00483] Brotheridge C. M., Grandey A. A. (2002). Emotional labor and burnout: Comparing two perspectives of “people work”. Journal of Vocational Behavior.

[B13-behavsci-16-00483] Burić I., Kim L. E., Hodis F. (2021). Emotional labor profiles among teachers: Associations with positive affective, motivational, and well-being factors. Journal of Educational Psychology.

[B14-behavsci-16-00483] Byron K. (2005). A meta-analytic review of work–family conflict and its antecedents. Journal of Vocational Behavior.

[B15-behavsci-16-00483] Carey S., Sutton A. (2024). Early childhood teachers’ emotional labour: The role of job and personal resources in protecting well-being. Teaching and Teacher Education.

[B16-behavsci-16-00483] Chang M. L. (2009). An appraisal perspective of teacher burnout: Examining the emotional work of teachers. Educational Psychology Review.

[B17-behavsci-16-00483] Choi S., Choi S. (2025). Authentic emotional displays and teacher well-being in early childhood education: The mediating role of affect states. Teaching and Teacher Education.

[B18-behavsci-16-00483] Clark S. C. (2000). Work/family border theory: A new theory of work/family balance. Human Relations.

[B19-behavsci-16-00483] Cohen S., Wills T. A. (1985). Stress, social support, and the buffering hypothesis. Psychological Bulletin.

[B20-behavsci-16-00483] Cole D. A., Maxwell S. E. (2003). Testing mediational models with longitudinal data: Questions and tips in the use of structural equation modeling. Journal of Abnormal Psychology.

[B21-behavsci-16-00483] Collie R. J., Shapka J. D., Perry N. E. (2012). School climate and social–emotional learning: Predicting teacher stress, job satisfaction, and teaching efficacy. Journal of Educational Psychology.

[B22-behavsci-16-00483] Côté S. (2005). A social interaction model of the effects of emotion regulation on work strain. Academy of Management Review.

[B23-behavsci-16-00483] Cumming T. (2017). Early childhood educators’ well-being: An updated review of the literature. Early Childhood Education Journal.

[B24-behavsci-16-00483] Demerouti E., Bakker A. B., Nachreiner F., Schaufeli W. B. (2001). The job demands-resources model of burnout. The Journal of Applied Psychology.

[B25-behavsci-16-00483] Deng H., Walter F., Lam C. K., Zhao H. H. (2017). Spillover effects of emotional labor in customer service encounters toward coworker harming: A resource depletion perspective. Personnel Psychology.

[B26-behavsci-16-00483] Diefendorff J. M., Croyle M. H., Gosserand R. H. (2005). The dimensionality and antecedents of emotional labor strategies. Journal of Vocational Behavior.

[B27-behavsci-16-00483] Edelwich J., Brodsky A. (1980). Burn-out: Stages of disillusionment in the helping professions.

[B28-behavsci-16-00483] Fredrickson B. L. (2001). The role of positive emotions in positive psychology. American Psychologist.

[B29-behavsci-16-00483] Frone M. R., Russell M., Cooper M. L. (1992). Antecedents and outcomes of work-family conflict: Testing a model of the work-family interface. Journal of Applied Psychology.

[B30-behavsci-16-00483] Fu C., Li F. (2025). The impact of work–family conflict on job and life satisfaction among construction workers: The mediating role of self-control ability. Sustainability.

[B31-behavsci-16-00483] Glavin P., Schieman S. (2012). Work–family role blurring and work-family conflict: The moderating influence of job resources and job demands. Work and Occupations.

[B32-behavsci-16-00483] Grandey A., Foo S. C., Groth M., Goodwin R. E. (2012). Free to be you and me: A climate of authenticity alleviates burnout from emotional labor. Journal of Occupational Health Psychology.

[B33-behavsci-16-00483] Grandey A. A. (2000). Emotional regulation in the workplace: A new way to conceptualize emotional labor. Journal of Occupational Health Psychology.

[B34-behavsci-16-00483] Grandey A. A., Gabriel A. S. (2015). Emotional labor at a crossroads: Where do we go from here?. Annual Review of Organizational Psychology and Organizational Behavior.

[B35-behavsci-16-00483] Grandey A. A., Melloy R. C. (2017). The state of the heart: Emotional labor as emotion regulation reviewed and revised. Journal of Occupational Health Psychology.

[B36-behavsci-16-00483] Greenhaus J. H., Beutell N. J. (1985). Sources of conflict between work and family roles. Academy of Management Review.

[B37-behavsci-16-00483] Gross J. J. (1998). The emerging field of emotion regulation: An integrative review. Review of General Psychology.

[B38-behavsci-16-00483] Grzywacz J. G., Marks N. F. (2000). Reconceptualizing the work-family interface: An ecological perspective on the correlates of positive and negative spillover between work and family. Journal of Occupational Health Psychology.

[B39-behavsci-16-00483] Hao D. (2024). An empirical study on the relationship between emotional labor and work performance among university teachers. Frontiers in Psychology.

[B40-behavsci-16-00483] Hobfoll S. E. (1989). Conservation of resources: A new attempt at conceptualizing stress. American Psychologist.

[B41-behavsci-16-00483] Hobfoll S. E. (2002). Social and psychological resources and adaptation. Review of General Psychology.

[B42-behavsci-16-00483] Hochschild A. R. (2012). The managed heart: Commercialization of human feeling.

[B43-behavsci-16-00483] Hu L. T., Bentler P. M. (1999). Cutoff criteria for fit indexes in covariance structure analysis: Conventional criteria versus new alternatives. Structural Equation Modeling: A Multidisciplinary Journal.

[B44-behavsci-16-00483] Jeon L., Buettner C. K., Grant A. A. (2018). Early childhood teachers’ psychological well being: Exploring potential predictors of depression, stress, and emotional exhaustion. Early Education and Development.

[B45-behavsci-16-00483] Jeung D.-Y., Kim C., Chang S.-J. (2018). Emotional labor and burnout: A review of the literature. Yonsei Medical Journal.

[B46-behavsci-16-00483] Johnson T., Kulesa P., Cho Y. I., Shavitt S. (2005). The relation between culture and response styles: Evidence from 19 countries. Journal of Cross-Cultural Psychology.

[B47-behavsci-16-00483] Karatepe O. M., Karadas G. (2014). The effect of psychological capital on conflicts in the work–family interface, turnover and absence intentions. International Journal of Hospitality Management.

[B48-behavsci-16-00483] Keller M. M., Becker E. S. (2021). Teachers’ emotions and emotional authenticity: Do they matter to students’ emotional responses in the classroom?. Teachers and Teaching.

[B49-behavsci-16-00483] Kernis M. H. (2003). TARGET ARTICLE: Toward a conceptualization of optimal self-esteem. Psychological Inquiry.

[B50-behavsci-16-00483] Kernis M. H., Goldman B. M. (2006). A multicomponent conceptualization of authenticity: Theory and research. Advances in Experimental Social Psychology.

[B51-behavsci-16-00483] Kuvaas B., Buch R., Weibel A., Dysvik A., Nerstad C. G. L. (2017). Do intrinsic and extrinsic motivation relate differently to employee outcomes?. Journal of Economic Psychology.

[B52-behavsci-16-00483] Kwon K. A., Ford T. G., Salvatore A. L., Randall K., Jeon L., Malek-Lasater A., Ellis N., Kile M. S., Horm D. M., Kim S. G., Han M. (2022). Neglected elements of a high-quality early childhood workforce: Whole teacher well-being and working conditions. Early Childhood Education Journal.

[B53-behavsci-16-00483] Landa I., English T. (2022). Variability in state authenticity predicts daily affect and emotion regulation. Emotion.

[B54-behavsci-16-00483] Lee M., Pekrun R., Taxer J. L., Schutz P. A., Vogl E., Xie X. (2016). Teachers’ emotions and emotion management: Integrating emotion regulation theory with emotional labor research. Social Psychology of Education.

[B55-behavsci-16-00483] Lenton A. P., Bruder M., Slabu L., Sedikides C. (2013). How does “being real” feel? The experience of state authenticity. Journal of Personality.

[B56-behavsci-16-00483] Li H., Liu J. (2025). The impact of work-family support and occupational stress on burnout among university teachers: The mediating role of psychological capital. Sage Open.

[B57-behavsci-16-00483] Luo Y., Zhang Y., Chen J., Lu G., Liu J., Chen C. (2025). The effect of emotional labor on young nurses’ job burnout: The mediating role of social support and emotional regulation. Nursing Open.

[B58-behavsci-16-00483] Luthans F., Luthans K. W., Luthans B. C. (2004). Positive psychological capital: Beyond human and social capital. Business Horizons.

[B59-behavsci-16-00483] Luthans F., Youssef C. M., Avolio B. J. (2006). Psychological capital: Developing the human competitive edge.

[B60-behavsci-16-00483] Marsh H. W., Hau K. T., Wen Z. (2004). In search of golden rules: Comment on hypothesis-testing approaches to setting cutoff values for fit indexes and dangers in overgeneralizing Hu and Bentler’s (1999) findings. Structural Equation Modeling.

[B61-behavsci-16-00483] Maslach C., Jackson S. E. (1981). The measurement of experienced burnout. Journal of Organizational Behavior.

[B62-behavsci-16-00483] Maslach C., Leiter M. P. (2016). Understanding the burnout experience: Recent research and its implications for psychiatry. World Psychiatry.

[B63-behavsci-16-00483] Maslach C., Schaufeli W. B., Leiter M. P. (2001). Job burnout. Annual Review of Psychology.

[B64-behavsci-16-00483] Mellors J., Gaspar T. (2025). Keeping it real? Authenticity and well-being at work. Annals of Tourism Research.

[B65-behavsci-16-00483] Michel J. S., Kotrba L. M., Mitchelson J. K., Clark M. A., Baltes B. B. (2011). Antecedents of work–family conflict: A meta-analytic review. Journal of Organizational Behavior.

[B66-behavsci-16-00483] Modan N. (2022). 45% of early childhood educators report high burnout, stress.

[B67-behavsci-16-00483] Moran C., Çoruk A. (2021). The relationship between emotional labor behavior and life satisfaction levels of primary school teachers. Trakya Eğitim Dergisi.

[B68-behavsci-16-00483] Muraven M., Shmueli D., Burkley E. (2006). Conserving self-control strength. Journal of Personality and Social Psychology.

[B69-behavsci-16-00483] Muthén L. K., Muthén B. (2017). Mplus user’s guide: Statistical analysis with latent variables, user’s guide.

[B70-behavsci-16-00483] Netemeyer R. G., Boles J. S., McMurrian R. (1996). Development and validation of work-family conflict and family–work conflict scales. Journal of Applied Psychology.

[B71-behavsci-16-00483] Rosco R. C., Yuayan A. P., Pilongo L. W. E. (2021). Public school teachers’ psychological capital, emotional labor, and stress index. ACADEME University of Bohol, Graduate School and Professional Studies Journal.

[B72-behavsci-16-00483] Ryan R. M., Deci E. L. (2000). Self-determination theory and the facilitation of intrinsic motivation, social development, and well-being. American Psychologist.

[B73-behavsci-16-00483] Schaack D. D., Le V.-N., Stedron J. (2020). When fulfillment is not enough: Early childhood teacher occupational burnout and turnover intentions from a job demands and resources perspective. Early Education and Development.

[B74-behavsci-16-00483] Schaufeli W. B., Bakker A. B. (2004). Job demands, job resources, and their relationship with burnout and engagement: A multi-sample study. Journal of Organizational Behavior: The International Journal of Industrial, Occupational and Organizational Psychology and Behavior.

[B75-behavsci-16-00483] Scott B. A., Lennard A. C., Mitchell R. L., Johnson R. E. (2020). Emotions naturally and laboriously expressed: Antecedents, consequences, and the role of valence. Personnel Psychology.

[B76-behavsci-16-00483] Sheldon K. M., Elliot A. J. (1998). Not all personal goals are personal: Comparing autonomous and controlled reasons for goals as predictors of effort and attainment. Personality and Social Psychology Bulletin.

[B77-behavsci-16-00483] Sheldon K. M., Goffredi R., Schlegel R. J. (2023). Self-concordant goal-striving as internalized motivation: Benefits beyond person-goal fit. Motivation Science.

[B78-behavsci-16-00483] Siu O. L., Bakker A. B., Jiang X. (2014). Psychological capital among university students: Relationships with study engagement and intrinsic motivation. Journal of Happiness Studies.

[B79-behavsci-16-00483] Skaalvik E. M., Skaalvik S. (2010). Teacher self-efficacy and teacher burnout: A study of relations. Teaching and Teacher Education.

[B80-behavsci-16-00483] Taxer J. L., Frenzel A. C. (2015). Facets of teachers’ emotional lives: A quantitative investigation of teachers’ genuine, faked, and hidden emotions. Teaching and Teacher Education.

[B81-behavsci-16-00483] Taxer J. L., Gross J. J. (2018). Emotion regulation in teachers: The “why” and “how”. Teaching and Teacher Education.

[B82-behavsci-16-00483] Ten Brummelhuis L. L., Bakker A. B. (2012). A resource perspective on the work-home interface: The work-home resources model. American Psychologist.

[B83-behavsci-16-00483] Toprak M., Tösten R., Elçiçek Z. (2024). Teacher stress and work-family conflict: Examining a moderation model of psychological capital. Irish Educational Studies.

[B84-behavsci-16-00483] Van den Bosch R., Taris T. W. (2014). The authentic worker’s well-being and performance: The relationship between authenticity at work, well-being, and work outcomes. The Journal of Psychology.

[B85-behavsci-16-00483] Wahid A.-S. A., Mohd I. H., Omar M. K. (2023). Psychological Capital (PSYCAP), emotional labour, and burnout in Malaysia: An overview. International Journal of Academic Research in Business and Social Sciences.

[B86-behavsci-16-00483] Wang H., Hall N. C., Taxer J. L. (2019). Antecedents and consequences of teachers’ emotional labor: A systematic review and meta-analytic investigation. Educational Psychology Review.

[B87-behavsci-16-00483] Wayne J. H., Matthews R. A., Odle-Dusseau H., Casper W. J. (2019). Fit of role involvement with values: Theoretical, conceptual, and psychometric development of work and family authenticity. Journal of Vocational Behavior.

[B88-behavsci-16-00483] White M. L., Wayne J. H., Casper W. J., Matthews R. A., Odle-Dusseau H., Jean E. L. (2024). The authentic self in work and family roles and well-being: A test of self-determination theory. Journal of Occupational and Organizational Psychology.

[B89-behavsci-16-00483] Wood A. M., Linley P. A., Maltby J., Baliousis M., Joseph S. (2008). The authentic personality: A theoretical and empirical conceptualization and the development of the Authenticity Scale. Journal of Counseling Psychology.

[B90-behavsci-16-00483] Xie D., Yang Y. (2025). When does authenticity benefit employee well-being: A relational framework of authenticity at work. Administrative Sciences.

[B91-behavsci-16-00483] Yan Z., Wang X., Li Y. (2022). Effects of psychological capital and person job fit on hospitality employees’ work family conflict, family work conflict and job performance: The moderating role of marital status. Frontiers in Psychology.

[B92-behavsci-16-00483] Yan Z., Zhang Z., Choo W. C. (2024). A meta analysis of antecedents and outcomes of psychological capital in hospitality and tourism. Journal of Hospitality Marketing & Management.

[B93-behavsci-16-00483] Yin H., Huang S., Chen G. (2019). The relationships between teachers’ emotional labor and their burnout and satisfaction: A meta-analytic review. Educational Research Review.

[B94-behavsci-16-00483] Yin H., Huang S., Lee J. C. K. (2017). Choose your strategy wisely: Examining the relationships between emotional labor in teaching and teacher efficacy in Hong Kong primary schools. Teaching and Teacher Education.

[B95-behavsci-16-00483] Yin H. B., Lee J. C. K. (2012). Be passionate, but be rational as well: Emotional rules for Chinese teachers’ work. Teaching and Teacher Education.

[B96-behavsci-16-00483] Zhang L., Yu S., Jiang L. (2020). Chinese preschool teachers’ emotional labor and regulation strategies. Teaching and Teacher Education.

[B97-behavsci-16-00483] Zhang Q., Yin J., Chen H., Zhang Q., Wu W. (2020). Emotional labor among early childhood teachers: Frequency, antecedents, and consequences. Journal of Research in Childhood Education.

[B98-behavsci-16-00483] Zheng J., Geng Y., Gao J., Xiang Q. (2024). Authenticity: Effective emotional labor strategies on teaching efficacy of university teachers in China. PLoS ONE.

[B99-behavsci-16-00483] Zhou Y. F., Nanakida A. (2023). Job satisfaction and self efficacy of in service early childhood teachers in the post COVID-19 pandemic era. Humanities and Social Sciences Communications.

[B100-behavsci-16-00483] Žiedelis A., Urbanavičiūtė I., Lazauskaitė-Zabielskė J. (2023). Family boundary permeability, difficulties detaching from work, and work-home conflict: What comes first during the lockdown?. Current Psychology.

